# Cytotoxic T lymphocyte-Associated Antigen +49G Variant Confers Risk for Anti-CCP- and Rheumatoid Factor-Positive Type of Rheumatoid Arthritis Only in Combination with CT60^*∗*^G Allele

**DOI:** 10.4061/2010/285974

**Published:** 2009-08-27

**Authors:** Bernadett Farago, Peter Kisfali, Lili Magyari, Noemi Polgar, Bela Melegh

**Affiliations:** Department of Medical Genetics and Child Development, University of Pecs, Pecs 7624, Hungary

## Abstract

Controversial observations have been published on the association of the cytotoxic T lymphocyte associated antigen gene's variants with rheumatoid arthritis (RA). After genotyping 428 patients and 230 matched controls, the prevalence of the CT60^∗^G allele was more frequent in RF- and/or anti-CCP-seropositive RApatients, compared to the healthy controls (*P* < .001). Regression analysis revealed that the CT60^∗^G allele is a possible predisposing factor for RA in these subgroups. No accumulation of the +49^∗^G allele was found among patients, and this variant was not found to correlate with RA. Assaying the possible genotype variations, the +49^∗^G-*C* *T*60^∗^G allelic combination was accumulated in seropositive RA-subtypes, and was associated with the risk of RA (OR = 1.73, *P* = .001 for the whole RA-population). Although the +49^∗^G allele did not mean a predisposition to RA alone, in combination with *C* *T*60^∗^G it, also conferred risk, suggesting that the +49*A*/*G* variant is associated with the risk of RA only in certain haplotypes.

## 1. Introduction

The cytotoxic T lymphocyte associated antigen (CTLA4) is a well-known cosignalling molecule of the B7 receptor immunoglobulin family. CTLA4 is expressed by CD4+, CD8+, T and B lymphocytes, and is located on the surface of the cell. Its expression and its density on the cell surface is affected by the activation of the T lymphocytes. Contrary to CD28, which transmits a stimulatory signal to the T cells by binding to B7-1 or B7-2 receptors on antigen presenting cells, CTLA4 is an inhibitory factor in T-cell activating systems [1]. 

 The *CTLA4* gene polymorphisms are reportedly associated with a variety of T cell-mediated autoimmune diseases such as type 1 diabetes mellitus [2, 3], systemic lupus erythematosus, Hashimoto thyroiditis [4], Addison disease [5], Grave's disease [6–8], multiple sclerosis [9–11], and celiac disease [12, 13]. Previous investigations on the association of *CTLA4* with RA [14–22] resulted in controversial outcomes. 

 Nistico et al. identified a +49A/G transition polymorphism of the leader sequence in exon 1, resulting in a threonine to alanine change at position 17 of the amino acid sequence [2]. In patients carrying the homozygous GG genotype, the hydrophobic alanine is inserted into the highly conservative sequence of the CTLA4 leader peptide. This slight alteration of the leader peptide may affect the intracellular transport of the CTLA4 protein and its availability on the cell surface [23]. As GG homozygotes have fewer functionally active CTLA4 molecules on the cell surface of the T lymphocytes, fewer B7-CTLA4 associations can form, leading to a less efficient CTLA4-mediated T cell inhibition. As a consequence, a significantly increased T cell proliferation can be observed, compared to AA homozygotes [24]. 

 Another polymorphism, the CT60 (A6230G) allele in the 3′-UTR region, encodes either a protective A variant or a predisposing G variant for autoimmune diseases [20]. The G allele is associated with lower mRNA levels of the soluble CTLA4 isoform. Both the +49A/G and the CT60A/G variants have been reported to confer risk for rheumatoid arthritis, however, the results are controversial [14], some findings affirm the role of the SNPs in RA [15, 17, 19, 20], while others could not reveal any association [16, 18, 21, 22]. The aim of the present study was to examine the distribution of these two SNPs in Hungarian RA patients separately and in haplotype combinations.

## 2. Materials and Methods

A total of 428 well-characterised RA patients (102 males and 326 females, mean age 54.9 ± 14.5 years), fulfilling the diagnostic criteria of the American College of Rheumatology [25] for rheumathoid arthritis were selected for the study. However, 230 (98 males, 132 females, mean age 57.4 ± 16.1 years) clinically healthy individuals were carefully selected as controls. None of the controls had any evidence or history of any major metabolic disorder, and a special care was taken to exclude patients with history of immunological diseases. All control subjects fit the criteria of RF- and anti-CCP-seronegativity, as a basic criterion in our studies [26–28]. All patients and controls were unrelated white Caucasians. The DNA samples of each group were obtained from a central pool governed by our department as part of the Central National Biobank Network of Hungary (http://www.biobank.hu/). The RA samples were originally collected in two medical centers, the University of Pecs and University of Debrecen, all other samples are from the University of Pecs. During the examination period, guidelines of the local Ethics Committee and the 1975 Helsinki Declaration were followed. 

 The sera from nonhaemolyzed blood of each RA patient was tested for the presence of RF and anti-CCP autoantibodies using the Rheumatoid Factor Screen ORG522S test by ORGENTEC Diagnostika GmbH (Mainz, Germany) and the enzyme immunoassay of Euro-Diagnostica (Malmö, Sweden), respectively. 

 The +49A/G (*GenBank rs231775*) and the CT60 A/G alleles (*GenBank rs3087243*) were examined by PCR RFLP methods. For the amplification of the target sequences, the following primers were designed: 5′-CTT GAG GTT GTC TTT TCG AG-3′ (forward) and 5′-TAC TAA ATA CCT GGC GCT CT-3′ (reverse) primers for +49G/A; and 5′-ATC TGT GGT GGT CGT TTT CC-3′ (forward) and 5′-TGG AAA CCA AAT GTG CTG AG-3′ (reverse) primers for CT60. The PCR amplifications were performed on MJ Research PTC 200 thermal cyclers using the following conditions: initial denaturation at 96°C for 3 minutes followed by 35 cycles of denaturation at 96°C for 45 seconds, annealing at 56°C for 45 seconds, and extension at 72°C for 45 seconds. The final extension was 10 minutes at 72°C. The amplified products were separated by agarose gel electrophoresis and visualized by ethidium bromide staining. 

 The amplicons were then digested by restriction endonucleases, +49A/G by *Bse XI* and CT60 by *Mae II*, as recommended by the manufacturer (Fermentas International Inc., Burlington, Ont, Canada). Both methods designed the amplicon contained an obligate restriction cleavage site allowing us to monitor the efficacy of the enyzme digestion. The digestion fragments were separated by agarose gel electrophoresis and visualized by ethidium bromide staining. 

 Statistical analysis was carried out using SPSS 11.5 for Windows. We performed binary logistic regression analyses and chi-square tests to reveal the possible associations between the genetic and serological characteristics.

## 3. Results

In this Hungarian cohort, we chose to evaluate the allele and genotype distribution of two variants of the *CTLA4* gene, in relation to the development of rheumatoid arthritis. The study comprised of 428 cases with RA and 230 healthy controls. Upon isolation of genomic DNA from all patients and controls, we performed genotyping using PCR-RFLP methods specific for *CTLA4* variants +49A/G and CT60. 

Table 1 presents our findings, the allele frequencies of the CT60 and +49A/G SNPs and of the +49*G-CT60*G haplotype in the patients and controls. The CT60*GG genotype and the CT60*G allele frequencies showed a significant increase in the RF- and/or anti-CCP-seropositive RA populations, while no difference was observed in the distribution of the +49A/G alleles compared to the healthy controls. All alleles studied here were in Hardy-Weinberg equilibrium in both the patient and control groups. 

 We also tested the frequencies of the possible haplotypic combinations and their significance. Comparing the prevalences of the +49*A-CT60*A, +49*A-CT60*G, +49*G-CT60*A, and +49*G-CT60*G combinations, the +49*G-CT60*G haplotype was found to accumulate in the entire RA patient population, and further analysis revealed that it was present at significantly elevated frequencies in the seropositive groups (Table 1). 

 The bivariate logistic regression analyses adjusted to age and gender revealed that the CT60*G and CT60*GG variants represent independent risk for the development of RF-, anti-CCP-seropositive and combined types of RA (Table 2). Similarly, the simultaneous presence of the CT60*G and +49*G alleles showed an approximately 1.5-fold increase in disease susceptibility considering the RF- and/or anti-CCP-seropositive cases. The +49*G variant alone was not found to confer risk for the disease.

## 4. Discussion

In the present study we genotyped two functional SNPs of the *CTLA4* gene in RA patients and matched controls, previously characterized for RF- and anti-CCP-seropositivity. The CT60*GG genotype and the CT60*G allele exhibited an accumulation in the entire RA group when compared with the healthy controls (33.9% versus 27.1% and 57.1% versus 48.5%). However, subsequent analysis of the data revealed a clear serology dependency: all seropositive groups of patients showed a significantly increased prevalence of the CT60*GG genotype and the CT60*G allele compared to the controls. On the other hand, no similar differences could be established between the seronegative patients and the control cases regarding the CT60*GG genotype- and CT60*G allele-distribution. This suggests that carrying the CT60*G variant does not mean predisposition for RF- and/or anti-CCP-seronegative subtypes of RA; however, it might confer susceptibility for RF- and/or anti-CCP-seropositive forms of the disease. 

 In RF- and anti-CCP-seropositive subjects the CT60*G allele represented an approximately 1.5-fold risk for the development of RA in our patient population. These results are in agreement with previous findings [19], reporting a slightly lower susceptibility (OR = 1.18, 95%CI: 1.07–1.31, *P* = .006) in Caucasian patients with anti-CCP-positive RA. Similarly, a Chinese cohort [29] demonstrated a susceptibility of OR = 1.41 (95%CI: 1.10–1.82, *P* = .005), considering the entire RA population with no regard to anti-CCP seropositivity. 

 It has been reported that the CT60*G variant is likely associated with the RF-seropositive form of RA [19]. So far no data has been published on samples seropositive, both for RF and anti-CCP. Our examinations revealed a risk of 1.58 (95%CI: 1.23–2.03, *P* < .001) in the double-seropositive group, which is similar to that observed in the subgroups characterized by seropositivity for either RF or anti-CCP (OR = 1.52, 95%CI: 1.20–1.93, *P* = .001; and OR = 1.58, 95%CI: 1.24–2.02, *P* < .001, resp.). 

 Based on the relatively high ratio of patients with the homozygous CT60*GG genotype, we hypothesize that the CT*60G is a susceptibility factor for RA in the Hungarian population. Furthermore, the CT60*GG homozygous state represents an approximately 2-fold risk for RA in the seropositive subsets of samples, compared to the risk of carrying the G allele in a heterozygous form. This may suggest a gene dose dependency in influencing disease susceptibility although the presence of two copies of the CT60*G variant does not mean an actual double risk, compared to a single copy of the variant. 

 Contrary to our findings and previous reports [19], some other studies on a Caucasian patient population failed to demonstrate any associations between the CT60*G variant and rheumatoid arthritis [22]. A likely explanation for this controversy is that cohorts including relatively small number of samples might fail to detect statistically significant differences, since the allele frequencies of the CT60 A and G variants are similar to each other in the general population. However, marginally significant differences regarding the susceptibility alleles may be detected in the patient group. Furthermore, the odds ratios found are relatively low (approximately OR = 1.5), therefore, it would not be surprising if studies with smaller sample size could not reveal any significant correlation between the possible risk alleles and the disease. Another explanation is that the above controversy might be due to heterogeneity across study populations, where a susceptibility allele identified in one population has no disease-association in another population. 

 Considering the +49A/G polymorphism, we could not detect any significant differences regarding the genotype and allele distributions of the RA and control samples (Table 1). Previous works [15–22] documented controversial outcomes on the possible association between this variant and RA. It is possible, that the +49A/G polymorphism does not increase disease susceptibility alone, but together with other predisposing factors, it increases the risk for developing RA, as some studies [17, 30, 31] demonstrated an association in HLA-DRB1*04 patients, while others [16, 22] found no association irrespective of the HLA-shared epitope status. A study on Chinese patients [29] suggested that CT60 is a possible independent causal variant in RA, and that the effect of the +49*G variant is dependent on the CT60*G allele, since when the +49*G allele is combined with the CT60*G in a haplotype, their effect is increased. We were not able to replicate these findings in our study (data not shown), although it is possible that the correlations found in Asian populations do not stand for Caucasians. 

 The exact molecular background of how the predisposing CT60*G variant affects CTLA4 function and thus T-cell activation is not fully understood. A recent report [20] indicated that the CT60 variant influences the splicing and production of soluble CTLA4, and that the CT60*G allele is associated with lower mRNA levels of the soluble CTLA4 isoform compared to the protective CT60*A variant. A smaller amount of functionally intact CTLA4 molecules will hinder the T-cell-attenuation, as the interaction between B7 receptor and CTLA4 is less efficient. A defect of this attenuating system may, therefore, be an important risk factor in the development of RA. Contrary to this, a recent study [32] could not support this hypothesis.

## 5. Conclusions

Although the molecular mechanisms through which the CT60 variant of *CTLA4* exerts its effect in the development of autoimmune diseases are yet to be clarified, it should be emphasized that the *CTLA4* CT60*G variant represents an 1.5-fold risk for the development of RA in the Hungarian sets of RF- and/or anti-CCP-seropositive rheumatoid arthritis patients. Since a relatively small number of studies have been conducted and controversial findings have been reported on the role of the +49A/G and the CT60 genetic variants in the development of RA, further investigations are required.

## Figures and Tables

**Table 1:** The distribution and frequencies of the CTLA4 CT60 and +49A/G genotypes and alleles, and the +49*G-CT60*G haplotype stratified by RF- and anti-CCP-seropositivity in patients versus controls.

	RA patients							Control

		RF		anti-CCP		RF + anti-CCP		

	Total	Positive	Negative	Positive	Negative	Positive-positive	Negative-negative	Total
	(*n* = 428)	(*n* = 324)	(*n* = 104)	(*n* = 310)	(*n* = 118)	(*n* = 286)	(*n* = 80)	(*n* = 230)

CT60								
AA (%)	84 (19.6)*	62 (19.1)*	22 (21.2)	59 (19.0)*	25 (21.2)	54 (18.9)*	17 (21.3)	64 (24.0)
GA (%)	199 (46.5)	143 (44.1)	56 (53.8)	131 (42.3)	68 (57.6)	122 (42.7)	47 (58.8)	109 (48.8)
GA+GG (%)	344 (80.4)	262 (80.9)	82 (78.8)	251 (81.0)	93 (78.8)	232 (81.1)	63 (78.8)	166 (78.4)
GG (%)	145 (33.9)*	119 (36.7)*	26 (25.0)	120 (38.7)*	25 (21.2)	110 (38.5)*	16 (20.0)	57 (27.1)
G (%)	489 (57.1)*	381 (58.8)*	108 (51.9)	371 (59.8)*	118 (50.0)	342 (59.8)*	79 (49.4)	223 (48.5)

+49A/G								
AA (%)	160 (37.4)	122 (37.7)	38 (36.5)	112 (36.1)	48 (40.7)	104 (36.4)	30 (37.5)	98 (42.6)
GA (%)	201 (47.0)	151 (46.6)	50 (48.1)	147 (47.4)	54 (45.8)	135 (47.2)	38 (47.5)	97 (42.2)
GA+GG (%)	268 (62.6)	202 (62.3)	66 (63.5)	198 (63.9)	70 (59.3)	182 (63.6)	50 (62.5)	132 (57.4)
GG (%)	67 (15.7)	51 (15.7)	16 (15.4)	51 (16.5)	16 (13.6)	47 (16.4)	12 (15.0)	35 (15.2)
G (%)	335 (39.1)	253 (39.0)	82 (39.4)	249 (40.2)	86 (36.4)	229 (40.0)	62 (38.8)	167 (36.3)

+49*G-CT60*G	182 (21.3)*	147 (22.7)*	35 (16.8)	147 (23.7)*	35 (14.8)	134 (23.4)*	22 (13.8)	62 (13.5)

*P < .05 in the prevalence of the AA and GG genotypes, the G allele, or the +49*G-CT60*G haplotype versus controls.

**Table 2:** The results of the binary logistic regression analyses considering the CT60*GG genotype, the CT60*G allele, and the +49*G-CT60*G haplotype in RA patients stratified by RF- and anti-CCP-seropositivity versus controls.

	RA Patients						

		RF		anti-CCP		RF + anti-CCP	

	Total	Positive	Negative	Positive	Negative	Positive-positive	Negative-negative

CT60*GG							
OR (95%CI)	1.56 (1.09–2.23)	1.76 (1.21–2.56)	1.01 (0.59–1.73)	1.92 (1.32–2.79)	0.82 (0.48–1.39)	1.90 (1.29–2.78)	0.76 (0.41–1.42)
*P*	.016	.003	.966	.001	.455	.001	.386

CT60*G							
OR (95%CI)	1.42 (1.13–1.78)	1.52 (1.20–1.93)	1.15 (0.83–1.60)	1.58 (1.24–2.02)	1.06 (0.78–1.46)	1.58 (1.23–2.03)	1.04 (0.72–1.49)
*P*	.003	.001	.410	< .001	.145	< .001	.845

+49*G - CT60*G							
OR (95%CI)	1.73 (1.27–2.37)	1.88 (1.36–2.61)	1.30 (0.83–2.04)	2.00 (1.44–2.76)	1.12 (0.71–1.75)	1.96 (1.41–2.73)	1.02 (0.61–1.73)
*P*	.001	<0 .001	.256	<0 .001	0.626	< .001	.931
